# Simulated Microgravity Influences Immunity-Related Biomarkers in Lung Cancer

**DOI:** 10.3390/ijms24010155

**Published:** 2022-12-21

**Authors:** Hend Baghoum, Hend Alahmed, Mahmood Hachim, Abiola Senok, Nour Jalaleddine, Saba Al Heialy

**Affiliations:** 1 College of Medicine, Mohammed Bin Rashid University of Medicine and Health Sciences, Dubai P.O. Box 505055, United Arab Emirates; 2 Meakins-Christie Laboratories, Research Institute of the McGill University Health Center, Montreal, QC H3A 2T5, Canada

**Keywords:** simulated microgravity, EMT, lung cancer, metastasis, tumor suppressor, biomarkers

## Abstract

Microgravity is a novel strategy that may serve as a complementary tool to develop future cancer therapies. In lung cancer, the influence of microgravity on cellular processes and the migratory capacity of cells is well addressed. However, its effect on the mechanisms that drive lung cancer progression remains in their infancy. In this study, 13 differentially expressed genes were shown to be associated with the prognosis of lung cancer under simulated microgravity (SMG). Using gene set enrichment analysis, these genes are enriched in humoral immunity pathways. In lieu, alveolar basal-epithelial (A549) cells were exposed to SMG via a 2D clinostat system in vitro. In addition to morphology change and decrease in proliferation rate, SMG reverted the epithelial-to-mesenchymal transition (EMT) phenotype of A549, a key mechanism in cancer progression. This was evidenced by increased epithelial E-cadherin expression and decreased mesenchymal N-cadherin expression, hence exhibiting a less metastatic state. Interestingly, we observed increased expression of *FCGBP*, *BPIFB*, *F5*, *CST1*, and *CFB* and their correlation to EMT under SMG, rendering them potential tumor suppressor biomarkers. Together, these findings reveal new opportunities to establish novel therapeutic strategies for lung cancer treatment.

## 1. Introduction

Space is known for lacking the gravity vector, which affects the body at organ, tissue, and cellular levels. Microgravity, a condition of apparent weightlessness, is a significant space stressor known to significantly impact human health, such as bone loss, muscle atrophy, and cardiac deconditioning [1,2]. The impact of microgravity on cancer cells has also been a growing focal point of interest in space and cancer research. Microgravity has been shown to suppress the activity of immune cells and disrupt multi-body systems, which may increase the risk of developing cancer [2,3,4]. Due to these obvious health issues, the microgravity environment has enabled researchers to study the biophysical mechanisms impacted by microgravity and helped discover therapeutics for neurodegenerative disorders, immunotherapies, and potentially better and more targeted anti-cancer therapies [4,5,6]. Numerous studies have well documented and reviewed the large implication that microgravity has on cellular progression, proliferation, and apoptosis in myriad tumor cell lines, including lung cancer [7,8,9,10,11,12]. However, many other studies involving microgravity are shown to induce changes in gene expression that are involved in cancer cell proliferation, metastasis, and survival, shifting the cells toward a less aggressive phenotype [13,14]. This may shed light on new understandings on tumor biology and diagnosis, hence highlighting microgravity as an innovative tool to discover novel targets in the hope of developing novel research approaches and to improving therapeutic strategies.

Lung cancer, with non-small cell lung cancer (NSCLC) as the most prominent type, is a leading cause of death, accounts for 12% of all cancers, and is the most commonly diagnosed cancer [15]. In studies involving microgravity, which may guide us to understand better the carcinogenic process, lung cancer cells were shown to lose their stemness after exposure to microgravity, thus affecting cancer cell growth and function [11]. A study by Ahn et al. showed that exposing lung cancer cells (A549) to microgravity induced rapid migration and proliferation. This was explained by the upregulated levels of matrix metalloproteases (MMP-2 and MMP-9) under microgravity, thus playing a crucial role in cancer invasion and migration [10]. In another study, Chung et al. found that simulated microgravity did not significantly affect lung cancer cell proliferation but rather increased migration as compared with the control group under normal gravity [16]. On the contrary, Chang et al. showed a reduction in migration in the A549 lung cancer cell line after 24  h of exposure to microgravity conditions [17]. Despite the significant effects of microgravity on cellular behavior, which comprises apoptosis, migration, and invasiveness, its effect on lung cancer is yet to be fully elucidated.

It is well established that epithelial cells undergo several biochemical changes to establish a mesenchymal phenotype that enhances apoptosis resistance, invasiveness, and migratory capabilities. Such a biological process is known as epithelial-to-mesenchymal transition (EMT), a hallmark of cancer progression [18]. The relationship of EMT to cancer progression has been well discussed. Tumors are known to activate EMT by upregulating transcription factors, expressing cell-surface proteins, and producing ECM-degrading enzymes. Once EMT is activated, the tumor cells lose their cell–cell adhesion and acquire migratory and invasive properties [19]. Only two studies have shown that simulated microgravity induces transient epithelial-to-mesenchymal transition (EMT) in keratinocytes [9]. In addition, such phenomena was highlighted in MCF-7 cells and HUVEC [20]; however, no studies have focused on the effect of microgravity on EMT in lung cancer.

Despite the many crucial discoveries made on the ability of microgravity to modulate tumorigenic and metastatic processes of cancer, the exact mechanisms driven by microgravity remain relatively unknown, hence rendering open questions regarding the adaptive changes that occur at the molecular level. In this study we have identified a signature of immune-related genes (*FCGBP*, *BPIFB1*, *F5*, *CFB*, *and CST1*) that significantly exhibited increased mRNA expression in A549 cells under the effect of simulated microgravity (SMG). In addition, we have evaluated the contribution of SMG in A549 cancer progression via EMT regulation. *FCGBP*, *BPIFB1*, *F5*, *CFB*, *and CST1* and their correlation to EMT key markers displayed a potentially less metastatic phenotype of lung cancer under SMG. The findings of this study provide a basis for future investigations that are required to identify underlying mechanisms, which will develop our understanding and aid in the development of novel therapeutic strategies for the treatment of lung cancer.

## 2. Results

### 2.1. Top Common Differentially Expressed Genes Identified in Lung Cancer Cells under Simulated Microgravity (SMG) Compared with Ground Gravity (GG)

**Figure 1:**
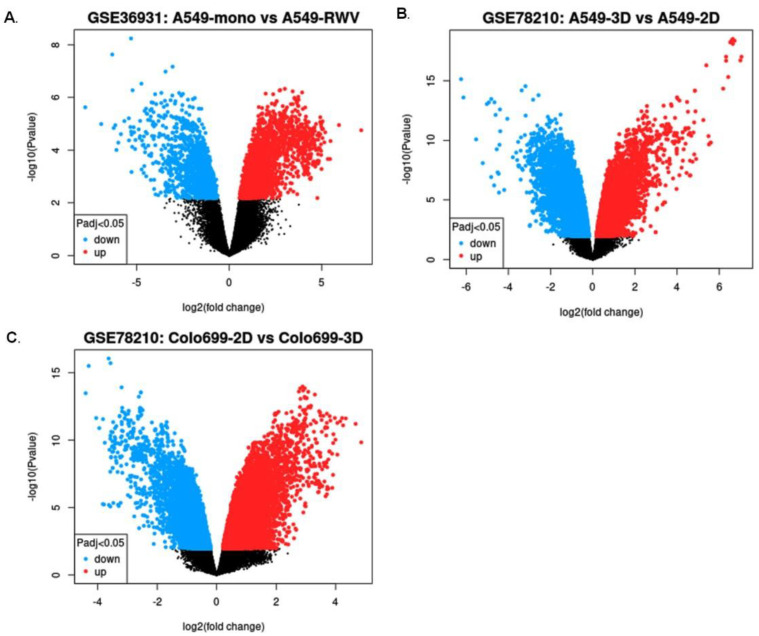
Volcano plot showing differentially expressed genes (DEGs) using GEO Omnibus. (**A**) Volcano plot showing DEGs under SMG and GG effects in A549 cells using (**A**) GSE78210 and (**B**) GSE36931 datasets. (**C**) Volcano plot showing DEGs under SMG and GG effects in Colo699 cell line using GSE78210 dataset. Red color indicates upregulated genes, while blue color indicates downregulated genes.

**Figure 2:**
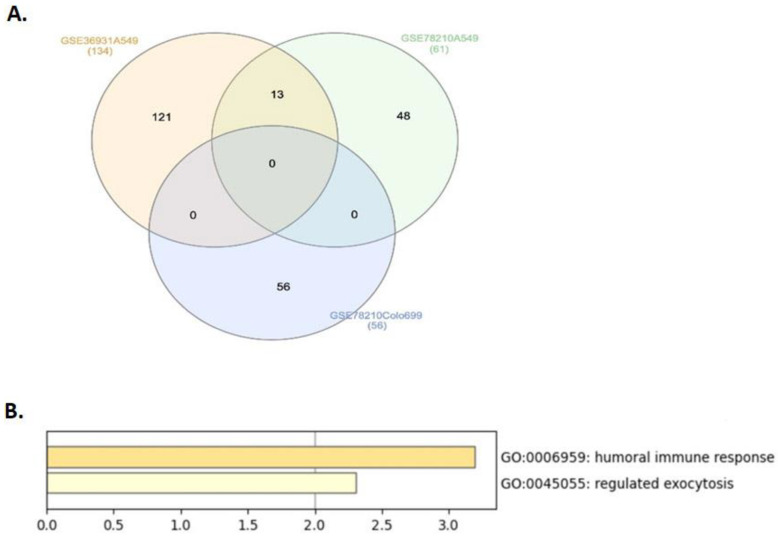
Thirteen commonly shared DEGs between GSE78210 and GSE36931 datasets in lung cancer. (**A**) Venn diagram showing 134 DEGs in GSE36931 (A549 cell line), 61 DEGs in GSE78210 (A549 cell line), and 56 DEGs in GSE78210 (Colo699 cell line) under SMG conditions as compared with GG. Out of the total identified 251 genes, 13 DEGs were found to be common between the two datasets, GSE78210 and GSE36931, in A549 cell line. Venn diagram was generated using InteractiVienn. Gene enrichment analysis was applied to the identified 13 genes. (**B**) Using Metascape (http://metascape.org; accessed on 11 November 2021), these genes are enriched in pathways related to humoral response and regulated exocytosis, using gene enrichment analysis.

To identify genes involved in lung cancer under simulated microgravity (SMG), we initially investigated genes that are differentially expressed under SMG compared with ground gravity (GG) for the human lung cancer using the publicly available Gene Expression Omnibus (GEO) database. Two datasets were chosen, GSE78210 and GSE36931, which include gene expression for two different human lung cancer cell lines, A549 and Colo699, cultivated in 2D and 3D cell culture conditions. Differences in genetic expression profiles between SMG and GG samples were presented in volcano plots (Figure 1). The identified differentially expressed genes (DEGs; Figure 2) for each dataset were divided into those that are upregulated in lung cancer (21 genes in A549 and 29 genes in Colo699 of GSE78210 dataset and 47 genes in A549 of GSE36931 dataset) and those that are downregulated in lung cancer (40 genes in A549 and 27 genes Colo699 of GSE78210 dataset and 87 genes in A549 of GSE36931 dataset), as represented in Table 1. Thirteen DEGs were significantly highly expressed in A549 cells of the GSE78210 and GSE36931 datasets in SMG compared with GG condition. The top most common genes were identified using the InteractiVienn web tool (Figure 2A). The candidate genes include *AZGP1*, *CFB*, *NOX1*, *VTCN1*, *AGR3*, *GDA*, *TCN1*, *CST1*, *F5*, *CEACAM6*, *BPIFB1*, *FCGBP*, and *BPIFA1*. To establish whether the identified DEGs are involved in common pathways, the candidate genes were uploaded to Metascape (http://metascape.org; accessed on 11 November 2021). Interestingly, these genes are enriched in pathways involving humoral immune response and regulated exocytosis (Figure 2B).

### 2.2. The Differentially Expressed Genes Correlated with Lung Adenocarcinoma Patients’ Clinical Prognosis

**Figure 3:**
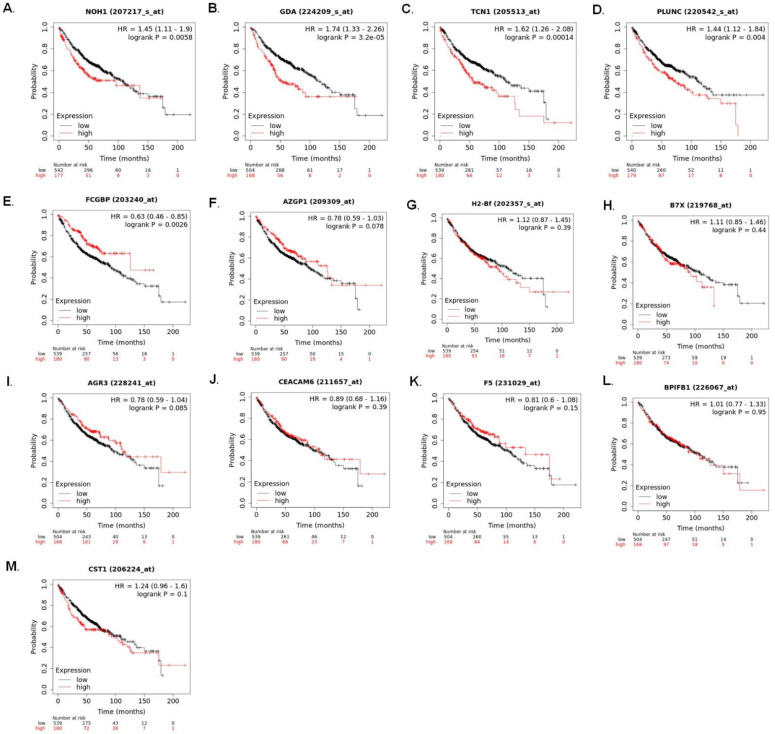
*FCGBP*, *CST1*, *F5*, *CFB*, and *BPIFB1* correlated to lung adenocarcinoma patient’s clinical prognosis. Kaplan–Meier survival curves of the mRNA expression levels of candidate genes: (**A**)*NOX1*; (**B**) *GDA*; (**C**) *TCN1*; (**D**) *PLUNC*; (**E**) *FCGBP*; (**F**) *AZGP1*; (**G**) *H2Bf*; (**H**) *B7X*; (**I**) *AGR3*; (**J**) *CEACAM6*; (**K**) *F5*; (**L**) *BPIFB1*; and (**M**) *CST1* in lung adenocarcinoma patients. HR: hazard ratio.

To evaluate the clinical prognostic value of the candidate genes on patients with lung adenocarcinoma, Kaplan–Meier plotter (http://www.kmplot.com/; accessed on 11 November 2021) was used to compare the overall survival (OS) of lung adenocarcinoma (LUAD) patients with high/intermediate versus low expression of candidate genes (Figure 3). High mRNA expression levels of NOX1 (hazard ratio (HR), 1.45; 95% confidence interval (CI), 1.11–1.9; *p* = 0.0058), GDA (HR, 1.74; 95% CI, 1.33–2.26; *p* = 3.2 × 10^−5^), TCN1 (HR, 1.62; 95% CI, 1.26–2.08; *p* = 0.00014), and BPIFA1 (HR, 1.44; 95% CI, 1.12–1.84; *p* = 0.0026) were observed to be significantly associated with poor prognosis (Figure 3A–D). On the other hand, high mRNA expression levels of FCGBP (HR, 0.63; 95% CI, 0.46–0.85; *p* = 0.0026) were associated with better prognosis and higher overall survival in LUAD patients (Figure 3E). There was no significant correlation between the mRNA expression levels of AZGP1 (HR, 0.78; 95% CI, 0.59–1.03; *p* = 0.078), CFB (HR, 1,12; 95% CI, 0.87–1.45; *p* = 0.39), VTCN1 (HR, 1.11; 95% CI, 0.85–1.46; *p* = 0.44), AGR3 (HR, 0.78; 95% CI, 0.59–1.04; *p* = 0.850, CEACAM6 (HR, 0.89; 95% CI, 0.68–1.16; *p* = 0.39), F5 (HR, 0.81; 95% CI, 0.6–1.08; *p* = 0.15), and BPIFB1 (HR, 1.01; 95% CI, 0.77–1.33; *p* = 0.95), as shown in Figure 3F–M.

### 2.3. Expression of FCGBP, BPIFB1, F5, CFB, and CST1 in A549 Cells Post-Simulated Microgravity

**Figure 4:**
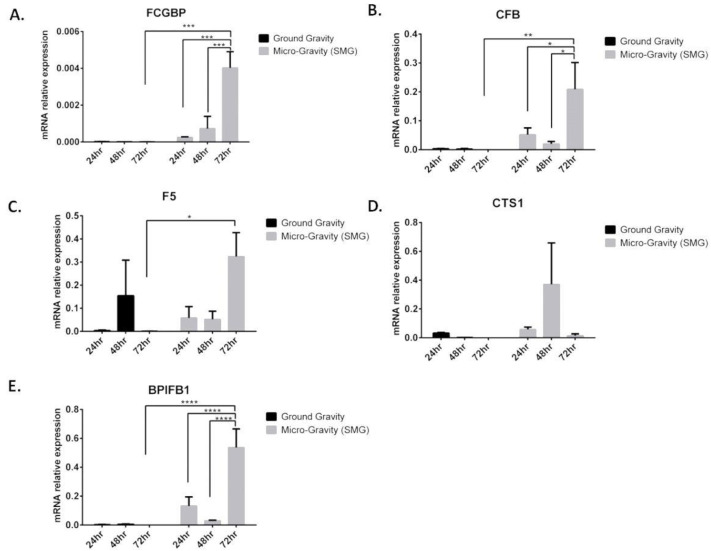
Upregulation of *FCGBP*, *CST1*, *F5*, *CFB*, and *BPIFB1* under SMG condition in vitro. mRNA expression levels of (**A**) *FCGBP*, (**B**) *CFB*, (**C**) *F5*, (**D**) *CTS1*, and (**E**) *BPIFB1*, quantified by qRT-PCR and normalized to *GAPDH* in A549 cells subjected to GG and SMG conditions for 24, 48, and 72 h. Bar graphs display results of 3 independent experiments (*n* = 3). * *p* < 0.05, ** *p* < 0.01, *** *p* < 0.001, and **** *p* < 0.0001.

To validate the expression of the in silico identified genes (*AZGP1*, *CFB*, *NOX1*, *VTCN1*, *AGR3*, *GDA*, *TCN1*, *CST1*, *F5*, *CEACAM6*, *BPIFB1*, *FCGBP*, and *BPIFA1*), in vitro application using qRT-PCR was performed. Interestingly, using qRT-PCR, our data displayed a significant upregulation of FCGBP mRNA expression, specifically at 48 and 72 h in A549 cells, post-SMG as compared with GG (Figure 4A). In addition, the mRNA expressions of CFB, F5, and BPIFB1 were significantly upregulated at 72 h post-SMG, and as compared with GG, in A549 cells (Figure 4B,C,E). A slight increase, although not significant, in mRNA expression was detected for CTS1 under SMG condition as compared with GG (Figure 4D). The expressions of *AZGP1*, *AGR3*, *GDA*, *VTCN1*, *BPIFA1*, *NOX1*, *CEACAM5*, and *TCN1* were undetermined for both SMG and GG (data not shown).

Overall, these data imply that the differentially expressed *FCGBP*, *F5*, *CFB*, and *BPIFB1* in lung cancer may play an important role in lung cancer progression.

### 2.4. Simulated Microgravity Reduces Cell Viability and Reverses Epithelial-to-Mesenchymal Transition in A549 Lung Cancer Cell Line

**Figure 5:**
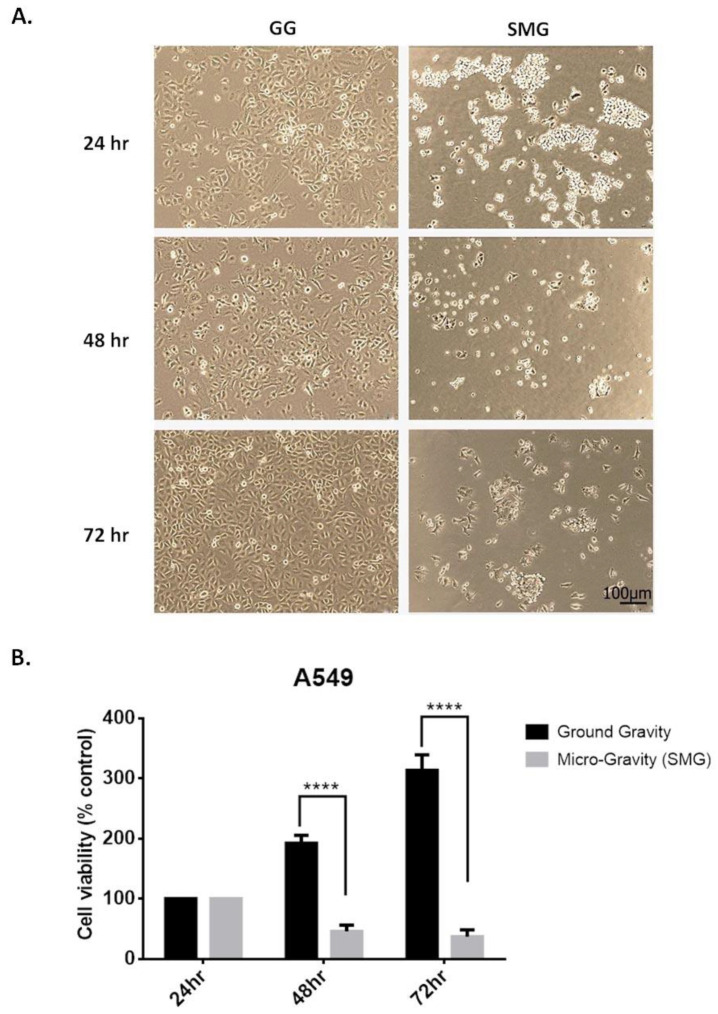
Simulated microgravity reduces cell viability and induces morphological changes of A549 cells in a time-dependent manner. (**A**) Representative micrographs of parental A549 cells (GG) and A549 cells subjected to SMG for 24, 48, and 72 h prior to seeding them back for 1hr under GG. Images were taken at 10X magnification. (**B**) Cell viability of A549 cells was assessed using trypan blue dye exclusion assay. Average cell viability of 3 independent experiments is displayed as percentage control. **** *p* < 0.0001.

To investigate whether SMG affects the proliferation potential of A549 cells, cell viability assay was performed using trypan blue exclusion assay. Our data revealed that SMG significantly induced a time-dependent decrease in cell proliferation (Figure 5B). The decrease in cell proliferation was significant mainly after 48 and 72 h post-SMG as compared with GG, which displayed an increase in cell proliferation of A549 cells. In parallel, a change in cellular morphology was detected in A549 cells under SMG. To assess these changes, cells were removed from the SMG condition, seeded onto 24-well plates, and observed under light microscopy after 1 h of SMG application. Interestingly, cells exposed to SMG revealed a granular, clumped morphology post-SMG. (Figure 5A).

**Figure 6:**
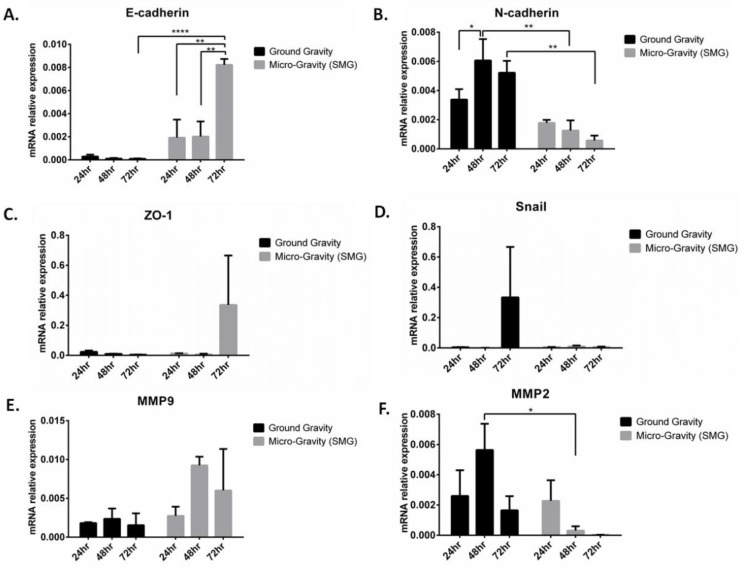
Downregulation of key EMT pathway genes in A549 under SMG condition. mRNA expression levels of (**A**) *E-cadherin*, (**B**) *N-cadherin*, (**C**) *ZO-1*, (**D**) *Snail*, (**E**) *MMP-9*, and (**F**) *MMP-2*, quantified by qRT-PCR and normalized to GAPDH in A549 cells subjected to GG and SMG conditions for 24, 48, and 72 h. Bar graphs display results of 3 independent experiments (*n* = 3). * *p* < 0.05, ** *p* < 0.01, and **** *p* < 0.0001.

To understand the obtained data on decreased cellular proliferation in A549, we evaluated the contribution of SMG on epithelial-to-mesenchymal transition (EMT) mechanism. EMT is a hallmark and an important mechanism that drives cancer progression and metastasis, thus enhancing its cell motility and invasive properties [19]. Key EMT markers (*E-cadherin*, *N-cadherin*, *ZO-1*, and *Snail*) and metalloproteinases *MMP-2* and *MMP-9* mRNA expression levels were measured by qRT-PCR in A549, under both GG and SMG (Figure 6). Upon SMG application, a significant increase of *E-cadherin* mRNA expression was observed, paralleled by a significant decrease of *N-cadherin* mRNA expression, at 48 and 72 h post-SMG (Figure 6A,B). A non-significant increase in *ZO-1* and *MMP-9* and a decrease of *Snail* transcription factor was detected upon SMG (Figure 6C–E). *MMP-2* was significantly decreased in A549 at 48 h post SMG (Figure 6F), indicating a less invasive form. Collectively, these data support a mesenchymal–epithelial transition (MET) phenotype induced by SMG in A549.

### 2.5. Epithelial-to-Mesenchymal Transition Pathway Correlates with the Expression of FCGBP, BPIFB1, F5, CFB, and CST1

**Figure 7:**
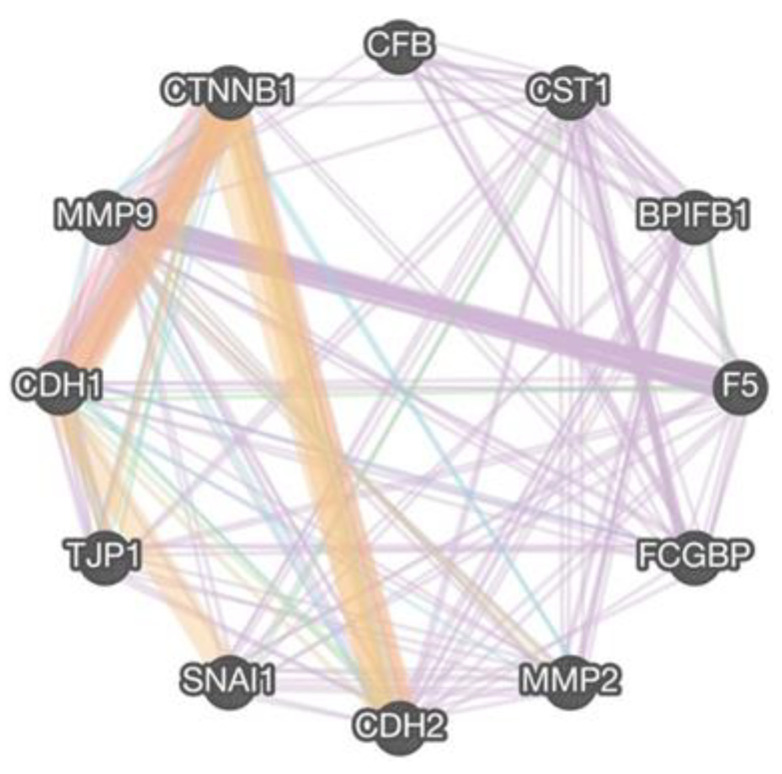
*FCGBP*, *CST1*, *F5*, *CFB*, and *BPIFB1* correlate with EMT pathway. Gene–gene interaction network of selected candidate genes; *FCGBP*, *CST1*, *F5*, *CFB*, and *BPIFB1* with EMT genes; *CDH1*, *CDH2*, *TJP1*, *CTNNB1*, and *SNAI1*, as well as cell-migration-related genes *MMP2* and *MMP9* generated by GeneMANIA (http://genemania.org/; accessed on 11 November 2021) to identify interactions between the candidate genes with EMT and cell migration markers. The different colors of the network edge indicate the bioinformatics methods applied: website prediction (orange), physical interactions (red), co-expression (purple), shared protein domains (brown), pathway (light blue), co-localization (dark blue), and genetic interactions (green).

To gain a further mechanistic insight into the potential role of the identified genes in A549 and their possible correlation to EMT under SMG condition, a gene-interaction analysis was conducted using GeneMANIA against EMT gene markers. Figure 7 shows the gene interactions plotted between the EMT genes E-cadherin (CDH1), N-cadherin (CDH2), TJP1, CTNNB1, SNAI1 (Snail), ZO-1, and β-catenin, respectively. Knowing their significant roles in cancer invasion and migration, the metalloproteinases MMP-2 and MMP-9 were also included in the analysis. The nodes identify co-expression patterns between the genes of varying strengths based on the thickness of the node. The candidate genes show a degree of co-expression between each other; BPIFB1 is co-expressed with CFB and CST1; FCGBP is co-expressed with F5, CST1, and CFB; CST1 is co-expressed with CFB, and F5 is co-expressed with CFB, CST1, and BPIFB1. Co-expression nodes were also identified between EMT genes and the candidate genes; CDH1 is co-expressed with FCGBP and F5; CDH2 is co-expressed with F5, CST1, BPIFB1, and FCGBP; CTNNB1 is co-expressed with CST1 and FCGBP; SNAI1 is co-expressed with CST1, F5, and BPIFB1; TPJ1 is co-expressed with CST1, FCGBP, and F5; CTNNB1 is co-expressed with CST1 and FCGBP; MMP2 is co-expressed with BPIFB1 and CST1; and MMP9 is co-expressed with F5, CST1, FCGBP, and CFB. These findings suggest a significant correlation between the novel identified genes and EMT markers under SMG condition, possibly related to EMT pathways.

## 3. Discussion

Lung cancer, one of the significant and worldwide threatening diseases, has recently gained very much attention in “Space research”. However, despite the advancement in this field, lung cancer progression and its response to treatment remain controversial due to the lack of definitive strategies of assessment that help in the prevention or treatment of cancer [21]. To our knowledge, this is the first report to identify a set of genes as a signature to lung cancer progression under a mechanical environment induced by simulated microgravity (SMG). We highlight the increased expression of immune-response-related genes *FCGBP*, *BPIFB*, *F5*, *CST1*, and *CFB*, and their correlation to epithelial-to-mesenchymal transition (EMT) under SMG, rendering them potential biomarkers of lung cancer progression. Herein, we also highlight the effect of induced SMG on EMT regulation, a hallmark of cancer progression [18,22], in supporting a mesenchymal-to-epithelial transition (MET) phenotype. Overall, this study offers the basis for the association of the identified genes with EMT; however, further investigations are required to unravel the exact mechanisms that aid in developing novel targeted therapy of lung cancer.

In this study we displayed thirteen genes (*AZGP1*, *CFB*, *NOX1*, *VTCN1*, *AGR3*, *GDA*, *TCN1*, *CST1*, *F5*, *CEACAM6*, *BPIFB1*, *FCGBP*, and *BPIFA1*) to be significantly expressed in lung cancer under simulated microgravity (SMG) as compared with ground gravity in silico. Our data showed that these differentially expressed genes are enriched in pathways related mainly to humoral immunity and regulated exocytosis. Interestingly, we also showed that these genes correlate to the overall survival of lung cancer patients, where some showed significant correlation to poor prognosis and others showed no potential correlation, except for *FCGBP*, which was significantly associated with better prognosis. FCGBP, or the Fc fragment of IgG binding protein, is a mucin-like glycoprotein found in body fluid and thought to play important roles in immunity and cancer [23,24]. This is in parallel with our findings, although the main function of FCGBP is still unclear [23]. This may be partly due to the contradictory roles it exhibits in different tumors [25]. For instance, it is shown that FCGBP is significantly associated with a better overall prognosis and disease-specific survivals in head and neck cancer, colon cancer, and osteosarcoma patients [25,26,27], while in ovarian and prostate cancers, the high expression of FCGBP was associated with poorer overall survival [28]. Of note, the role of FCGBP role has been attributed to immune defense mechanisms, anti-inflammatory responses, as well as cell protection [29,30], hence rendering it an essential prognostic marker [31].

Using in vitro applications, we have evaluated the expression of the identified genes in lung cancer and under SMG condition via a 2D clinostat. There is a broad selection of clinostat types and other microgravity platforms that have been developed. These include 1/2/3 D clinostat systems, random positioning machines equipped with slide flasks (mainly for thyroid cancer cells), and NASA-developed rotating wall vessel. Each is designed to serve a certain research objective, where some are designed to cultivate adherent or suspension cells, and others are used for online measurement of kinetic responses. Of note, the clinostat is one of the simplest and most adaptable platforms used by different experimental applications. In principle, the 2D clinostat is characterized by a rotation axis that rotates continuously at an adjusted constant speed and in a direction that is perpendicular to the direction of the Earth’s gravity vector, hence creating centrifugal forces that mimic real microgravity [14,32,33]. Interestingly, of the thirteen genes, *FCGBP*, *BPIFB*, *F5*, *CST1*, and *CFB* expressions significantly increased in response to 2D clinostat-induced SMG in A549 cells. The expressions of *FCGBP* and *BPIFB* were the most affected, showing significant increase at all assessed time points: 24, 48, and 72 h post-SMG. Similar to FCGBP, BPIFB1 is known to contribute to the innate immunity responses [34]. The BPI fold containing family B member 1 protein (BPIFB1), which is primarily produced by airway epithelia, has proved to participate in host-defense mechanisms, along with bactericidal and anti-inflammatory effects [35]. In respiratory diseases, BPIFB1 is shown to exhibit anti-tumor and anti-metastatic effects; however, the exact mechanisms remain unclear, warranting further investigation [34,36]. One study by Wei et al. found that BPIFB1 inhibits migration and invasion of nasopharyngeal carcinoma [37].

Given that, it was found that mutations occurring in *BPIFB1* promote the risk of lung cancer, and its downregulation leads to poor prognosis in lung cancer patients [38,39]. In addition to the role of FCGBP and BPIFB, the complement factor B (CFB) and the chimeric tumor suppressor 1 (CST1) are shown to exert protective roles in cancer. For instance, it was reported that high expression of *CFB* was associated with increased patient overall and disease-free survival in lung cancer patients [40]. Furthermore, the complement system serves as the first line of defense against pathogens, serving as a major component of both the innate and acquired immune systems [41]. As for CST1, different studies highlighted its interesting properties, such as its ability to suppress cell growth, induce apoptosis, and its resistance to the inactivation by oncogenic forms of p53, rendering it an attractive, yet alternative therapeutic target of wild-type p53-resistant human tumors [42].

In addition to the increased expression of *FCGBP*, *BPIFB1*, *F5*, *CFB*, *and CST1*, our data interestingly displayed two cellular-related phenotypes of A549 cells under SMG—a morphology change into aggregates and clump-shaped cells and a significant decrease in the proliferation rate post-SMG. This is in parallel with some other studies, where cells that were exposed to SMG exhibited small clumps or multi-layered cell aggregates [43,44]. These morphological differences are mirrored by the concomitant dramatic functional changes in cellular processes, part of which is the EMT. EMT is a hallmark of the metastatic process, associated with the escape of the immune surveillance and invasion of the vasculature, hence allowing the cells to disseminate to secondary organs [19,22]. In principle, malignant epithelial cells undergo an EMT mechanism at the primary stages of the tumor development, where these cells tend to express mesenchymal properties, exhibiting increased motility that facilitates their escape from the primary niche, whereas the metastatic cells formed at the secondary side display less dedifferentiated properties as compared with their corresponding primary tumors. Herein, a MET (mesenchymal-to-epithelial) process is part of such a progression of metastatic tumor formation. However, that does not neglect the participation of EMT at different stages of the cancer progression due to that fact that cancer cells at secondary sites will either continue to grow and proliferate or undergo dormancy [45,46]. Such multiple gene coordination and appearance of specific migratory markers that regulates the metastatic progression of cancer cells may result in different outcomes upon SMG exposure [47]. For instance, under microgravity conditions, different breast cancer cells exhibited different morphologies, cell-adhesion, and migratory properties upon exposure to microgravity [5]. Of note, the main characteristic of EMT is the loss of the cell adhesion molecule, E-cadherin, thus affecting the quiescence of the cells’ integrity. On the other hand, the increase of the neural cadherin, N-cadherin, leads to cell adhesion alterations. This is induced mainly by transforming growth factor (TGF-β), which activates pleiotropically expressed transcription factors, such as Snail, Twist, and Zeb proteins and other signaling pathways implemented in metastatic pathways [18,48,49]. Herein, we report the enhancement of the epithelial marker *E-cadherin* (*CDH1*) and the downregulation of the mesenchymal *N-cadherin* (*CDH2*) mRNA levels, key markers of EMT [49]. This implies that in combination with our data on the reduced proliferation rate of A549, the cells are exhibiting a less metastatic state. Interestingly, the altered expression of key EMT markers was also accompanied with a significant downregulation of the matrix metalloproteinase *MMP-2*, which is a major component of the basement membrane that normally segregates the epithelial layer from the surrounding mesenchyme, thus leading to the loss of the basement membrane [50]. This is in parallel with a study by Chang et al., where they reported their findings of reducing the metastatic potential of human lung adenocarcinoma cells through the alteration of MMP2 expression [17]. In general, the role of MMPshave been well implicated in malignancies progression, including metastasis, where a reduced MMP (MMP-2 and MMP-9) expression and enzymatic activity is a characteristic of a less metastatic phenotype [50,51,52]. Having the significant role of EMT in metastasis, our data highlight a significant correlation of *FCGBP*, *BPIFB*, *F5*, *CST1*, and *CFB* with EMT gene markers as well as cell-migration-related genes MMP2 and MMP9. Interestingly, Xiong et al. have shown FCGBP to be a main regulator of EMT in the gall bladder [53]. This signifies that the identified genes may serve as potential biomarkers for future studies that can predict and further understand the progression lung cancer.

Collectively, our findings classify the identified genes as key regulators of EMT, promoting a potential enhancement of a less metastatic phenotype via a mesenchymal-to-epithelial transition (MET) of lung cancer cells.

## 4. Materials and Methods

### 4.1. Datasets to Identify Common Differentially Expressed Genes (DEGs) in Lung Cancer Cell Lines Exposed to Simulated Microgravity

Relevant datasets from publicly available Gene Expression Omnibus (GEO) database were extracted for initial analysis. This database is used as a publicly available functional genomics analysis of high throughout gene expression data and microarrays. The datasets selected fulfilled the following criteria: datasets using human lung cancer cell lines only, studies that include matching controls in ground gravity (GG) conditions, datasets with defined classification of lung cancer cell lines, and datasets with human lung cancer cell gene expression using microarray. Two datasets fit this criteria, GSE78210 and GSE36931. In total, 25 samples were included in the studies, and 14 lung cancer cell line samples in simulated microgravity (SMG) were compared with 11 GG controls, as shown in Table 2 (https://www.ncbi.nlm.nih.gov/geo/; accessed on 11 November 2021).

### 4.2. GEO2R Gene Set Enrichment Analysis to Generate DEGs in Each Dataset

GEO2R (https://www.ncbi.nlm.nih.gov/geo/geo2r; accessed on 11 November 2021), an interactive online tool used to compare GEO series, was used in each dataset to group and identify upregulated and downregulated genes in SMG conditions compared with GG conditions. The genes with *p*-values less than 0.05 and fold change greater than 2 were selected as differentially expressed genes DEGs in SMG conditions. Consequently, the DEGs of each dataset were intersected with each other, and common genes were identified.

### 4.3. Quantification of Clinicopathological Involvement of the Identified Genes

Next, the resulting short-listed genes were validated using Kaplan–Meier Plotter (http://www.kmplot.com/; accessed on 11 November 2021), an online public database evaluating the effect of selected genes on patient clinical outcomes. Overall survival (OS) was defined as the duration from the time of diagnosis (in months) until death. Gene expression data and the survival information are derived from the Gene Expression Omnibus (GEO), The Cancer Genome Atlas (TCGA), and European Genome-phenome Atlas (EGA). Patients were categorized into two groups: (1) high expression (with TPM values above the upper quartile) and (2) low/medium expression (with TPM values below the upper quartile). Kaplan–Meier plots were used to compare OS of lung adenocarcinoma (LUAD) patients with high/intermediate versus low expression of candidate genes.

### 4.4. Enriched Ontology Clustering for the Identified Genes

To explore whether the identified genes share common pathways, Gene Ontology (GO) pathway enrichment analysis for the identified genes was performed using Metascape web tool (https://metascape.org/; accessed on 11 November 2021) for comprehensive gene list annotation and analysis resource alongside GEO2R.

### 4.5. Cell Line and Cell Culture

Human adenocarcinoma alveolar basal epithelial (A549) cell line, widely used as a model for lung adenocarcinoma, were grown in Roswell Park Memorial Institute Medium (RPMI)-1640 medium supplemented with 10% fetal bovine serum (FBS; Sigma, St. Louis, MO, USA) and 100 units/mL of penicillin/streptomycin (P/S; Sigma, St. Louis, MO, USA). The cell line was cultured in a 37 °C humidified incubator in an atmosphere of 5% CO_2_. When the cells reached confluence, they were harvested using 0.5% trypsin, centrifuged at 100× *g* for 5 min, and used when required.

### 4.6. Microgravity

Lung cancer cells (A549) were cultured in a 2D clinostat system mimicking the near-zero gravity environment (microgravity). This Rotary Culture Max (RCMW™; Synthecon^®^ Inc—Houston, TX, USA) is a bioreactor equipped with cell culture vessel incorporating a microporous perfusion core and an *in-line* oxygenator system, which provides nutrient and external gassing of the media, hence insuring proper gas exchange and a low-shear cell culture environment. The chamber was packed with the culture media containing the cells. This chamber horizontally rotated around one axis perpendicular to the force of gravity at a speed of 10 rpm. The 2D clinostat was placed inside the 37 °C humidified incubator in an atmosphere of 5% CO_2_. A549 cells were harvested post-simulated microgravity (SMG) at 24, 48, and 72 h.

To create a comparable lung cancer environment, A549 cells were cultured in culture plates at GG, in a non-rotated or static condition and were kept in proximity of the device inside the humidified incubator (37 °C). These cells are referred to as the control group and were also harvested at 24, 48, and 72 h.

### 4.7. Cell Viability Assay

For the GG conditions, A549 cells were seeded in 24-well cell culture plates at a density of 3.0 × 10^4^ cells per 500 µL. Cells were harvested by trypsinization at 24, 48, and 72 h following seeding, then centrifuged at 200× *g* for 5 min. Obtained cell pellets were reconstituted in culture media.

For the SMG conditions, A549 cells were seeded in the 2D clinostat rotating system at a density of 6.0 × 10^4^ cells per 1 mL. Cells were harvested at 24, 48, and 72 h following SMG exposure, then centrifuged at 200× *g* for 5 min. Obtained cell pellets were reconstituted in culture media.

Trypan blue (Sigma, USA) dye exclusion assay was performed. Cells count was determined using the CellDrop™ Automated Cell Counter.

### 4.8. Quantitative Real-Time Polymerase Chain Reaction (qRT-PCR)

Gene expression levels (mRNA levels) of the in silico identified genes (*AZGP1*, *CFB*, *NOX1*, *VTCN1*, *AGR3*, *GDA*, *TCN1*, *CST1*, *F5*, *CEACAM6*, *BPIFB1*, *FCGBP*, and *BPIFA1*) along with EMT gene markers (Table 3) were also determined in A549 cells by qRT-PCR. Briefly, extraction of total RNA from cells was performed using RNEasy Mini Kit (QIAGEN, Hilden, Germany) at 24, 48, and 72 h at GG and after exposure to SMG by following the manufacturer’s protocols. One μg of total RNA was reverse-transcribed to a single stranded complementary DNA (cDNA) in a 20 µL reaction volume using iScript™ cDNA synthesis kit (Thermo, Waltham, MA, USA). qRT-PCR was performed using PowerUp™ Sybr™ Green master mix (Thermo, USA) in a Quant Studio 5 pcr machine (Thermo, USA). PCR amplification steps were as follows: an initial denaturation step at 95 °C for 3 min, annealing temperature of the target gene for 30 s, and then 72 °C for 30 s. The fluorescence threshold cycle value was obtained for each gene. ∆∆Cq method was used to calculate the relative fold change in gene expression after normalization to the housekeeping gene, Glyceraldehyde 3-phosphate dehydrogenase (GAPDH).

### 4.9. Gene–Gene Interaction Analysis

Data from in vitro qRT-PCR was implemented in gene–gene interaction analysis using in silico webtool GeneMANIA (http://genemania.org/; accessed on 11 November 2021), a network algorithm for predicting gene functions using a large set of functional association data. This was conducted to further evaluate interactions between/among the selected candidate genes; *FCGBP*, *CST1*, *F5*, *CFB*, and *BPIFB1* with EMT genes; *CDH1*, *CDH2*, *TJP1*, *CTNNB1*, and *SNAI1*, as well as cell-migration-related genes, *MMP2* and *MMP9*.

### 4.10. Statistical Analysis

GraphPad Prism software was used to perform statistical analysis. Results are expressed as individual data or as mean ± standard deviation (SD). Student’s *t*-test was used to compare various groups. Difference between groups were assessed by one-way anova of variance (ANOVA). *p*-values were determined and values of *p* < 0.05, *p* < 0.01, *p* < 0.001 ( *, **, and ***, respectively) were considered as significant. All experiments were performed in triplicate (*n* = 3).

## Figures and Tables

**Table 1:** Total genes that are differentially expressed at SMG compared with GG in lung cancer cell lines A549 and Colo699. Each dataset had fold changes adjusted to extract the top 5% of DEGs. For GSE36931, fold change (FC) was adjusted to include only genes with an FC greater than or equal to 4 or less than or equal to −4. For GSE78210, FC was adjusted to include only genes with an FC greater than or equal to 3 or less than or equal to −3.

Dataset	Lung Cancer Cell Line	Log2 (Fold Change)	Total DEGs between SMG-Exposed Lung Cancer Cells and Controls	Genes Upregulated in SMG	Genes Downregulated in SMG
GSE36931	A549	4 or −4	134	47	87
GSE78210	A549	3 or −3	61	21	40
GSE78210	Colo699	3 or −3	56	29	27

**Table 2:** Details of Datasets Extracted from Gene Expression Omnibus (GEO) used for Initial Identification of DEG Between Simulated Microgravity (SMG) and Ground Gravity (GG) Lung Cancer cells.

Geo ID	Title	Lung Cancer Cell Line(s)	Total	Simulated Microgravity Condition	Ground Gravity Condition
GSE 36931	Rotating wall vessel (RWV) grown A549s compared with monolayer grown A549s	A549	10	5	5
GSE78210	3D cultivation of NSCLC cell lines alters gene expression of key cancer-associated signaling pathways	A549 and Colo699	15	9	6
Total	2		25	14	11

**Table 3:** List of human primers. *NOX1*, NADPH Oxidase 1; *GDA*, Guanine Deaminase; *TCN1*, Transcobalamin 1; *FCGBP*, Fc Gamma Binding Protein; *BPIFA1*, BPI Fold Containing Family A Member 1; *AZGP1*, Alpha-2-Glycoprotein 1; *CFB*, Complement Factor B; *VTCN1*, V-Set Domain Containing T Cell Activation Inhibitor 1; *AGR3*, Anterior Gradient 3; *CTS1*, chimeric tumor suppressor 1; *F5*, Coagulation Factor V; *CEACAM6*, CEA Cell Adhesion Molecule 6; *BPIFB1*, BPI Fold Containing Family B Member 1; *ZO-1*, Zonula occludens protein 1; *MMP*-9, Matrix metalloproteinase 9; *MMP-2*, Matrix metalloproteinase 2; and *GAPDH*, Glyceraldehyde 3-phosphate dehydrogenase.

PRIMER NAME	FORWARD PRIMER	REVERSE PRIMER
NOX1	GGAACTCTTGGGGTAGGTGT	GCATCCACAAACAGGAAAAC
GDA	AGAGAGTCCCGCTGCGTCT	GGCCAGTTTTTCCTGTTGAG
TCN1	GGTACACTGTTGGAGAGATGA	CGCTGGTTCCCCTGTTATAG
FCGBP	AGCCATGGGTGCCCTATGG	GGCTGAGGATGGAGACTGAA
BPIFA1	GGACAGCTGCTGAGACCTC	GGGCTGGATTCACATTCAAG
AZGP1	GCAGACACAATGGTAAGAATGG	AGGTCAGAGAGTAACGACCATC
CFB	GGTCTGGAGTTTCAGCTTGG	TCTTGGAGAAGTCGGAAGGA
VTCN1	TCAGCCAGTACCCAGATACG	TGCTCCAGCCAGAATAATGA
AGR3	GGCCAAGTCAGCTTCTTCTG	GTCATCTCCCCATCCTCTTG
CTS1	CAGCTTTGTGCTCTGCCTCT	CACGCTGTACCCACTCATCA
F5	GAGCAGGAAAGGAAGCATGT	TCGGTAGCTCCAACTGATGC
CEACAM6	CAGAAGGAGGAAGGACAGCA	CACCACTGCCAAGCTCACTA
BPIFB1	GTCTGGCATCCTGCACTTG	GACTGAGGGTGGCTTGGAT
E-Cadherin	CAGAAAGTTTTCCACCAAAG	AAATGTGAGCAATTCTGCTT
N-Cadherin	GGTGGAGGAGAAGAAGAAGACCAG	GGCATCAGGCTCCACAGT
Snail	CTTCCAGCAGCCCTACGAC	CGGTGGGGTTGAGGATCT
β-catenin	AGGGATTTTCTCAGTCCTTC	CATGCCCTCATCTAATGTCT
ZO-1	CAGCCGGTCACGATCTCCT	TCCGGAGACTGCCATTGC
MMP-9	TTGACAGCGACAAGAAGAAGTGG	GCCATTCACGTCGTCCTTAT
MMP-2	TTGACGGTAAGGACGGACTC	ACTTGCAGTACTCCCATCG
GAPDH	TGGTGCTCAGTGTAGCCCAG	GGACCTGACCTGCCGTCTAG

## Data Availability

All data are contained within the manuscript.
